# Supplementary material for: Susceptibility-Weighted MRI for Predicting NF-2 Mutations and S100 Protein Expression in Meningiomas
Source: Diagnostics (Basel). 2024 Mar 31;14(7):748. doi: 10.3390/diagnostics14070748 (PMC11012050; doi:10.3390/diagnostics14070748)
Supplement: Supplementary file 1 [file diagnostics-14-00748-s001.zip › diagnostics-2868958-supplementary.pdf]

Supplementary Document

First order textural features of SWI and morphological features were compared among subgroups of S100 protein expression and NF-2 copy number loss datasets. The results are summarized in Table S2-S4 and Figure S1-S6.

Table S1. Comparison of Textural Features for Meningiomas with and without S100 Protein Expression

**Features**	**P value**	**S100 (+)**	**S100 (-)**
10Percentile	0.105	126.89 (-69.76 – 172.16)	117.02(-76.24 – 167.87)
90Percentile	0.023	192.58 (8.33 – 264.09)	188.96 (41.59 – 240.46)
Energy (x10^9)	0.273	0.85 (0.16 – 4.49)	0.96 (0.52 – 5.92)
Entropy	0.620	4.69 (3.76 – 5.87)	4.67 (3.85 – 5.95)
InterquartileRange	0.974	38.34 (19.57 – 186.65)	39.36 (17.47 – 170.64)
Kurtosis	0.378	8.63 (1.85 – 18.14)	9.30 (1.88 – 27.61)
Maximum	0.078	257.74 (147.76 – 597.59)	273.83 (203.79 – 640.10)
MeanAbsoluteDeviation	0.697	28.19 (13.82 – 85.29)	27.88 (14.15 – 83.22)
Mean	0.035	166.26 (-40.08 – 213.00)	154.56 (-34.04 – 190.69)
Median	0.037	173.05 (-50.43 -216.22)	161.30 (-54.09 – 194.45)
Minimum**	**<0.001**	-78.36 (-130.21- 36.67)	-97.44 (-143.72 – 2.85)
Range	0.009	331.82 (210.25 – 727.90)	376.19 (215.74 – 745.15)
RobustMeanAbsoluteDeviation	0.985	16.83 (8.48 – 71.60)	16.02 (7.56 – 68.10)
RootMeanSquared	0.034	467.29 (262.37 – 515.24)	455.76 (270.90 – 491.57)
Skewness	0.285	-1.74 (-3.24 – 1.88)	-1.76 (-4.03 – 1.76)
Uniformity	0.811	0.05 (0.01 – 0.09)	0.05 (0.01 – 0.09)
Variance	0.382	1561.03 (378.33 – 9236.16)	1808.95 (453.96 – 8945.02)

The data are presented as median values and ranges.
Textural features were calculated using Pyradiomics.
A statistically significant P value is denoted as ** (0.0027 after Bonferroni correction, 0.05 divided by 18, the number of first-order textural features).

Table S2. Comparison of Textural Features for Meningiomas with and without NF-2 Copy Number Loss

Features	P value	NF-2 Loss	NF-2 Intact
10Percentile	0.806	118.28 (-75.24 – 173.65)	120.77 (-63.28 – 166.17)
90Percentile	0.649	195.86 (33.02 – 264.09)	199.56 (124.79 – 276.31)
Energy (x10^9)	0.904	0.91 (0.77 – 5.91)	0.97 (0.55 – 5.16)
Entropy	0.671	4.70 (3.76 – 5.77)	4.75 (3.76 – 6.04)
InterquartileRange	0.746	39.96 (19.57 – 118.28)	42.17 (19.42 – 186.14)
Kurtosis	0.916	8.58 (2.19 – 23.24)	8.50 (1.85 – 23.68)
Maximum	0.008	264.03 (193.09 – 459.52)	292.07 (206.78 – 687.80)
MeanAbsoluteDeviation	0.867	28.90 (13.82 – 64.96)	28.74 (13.95 – 90.28)
Mean	0.904	159.97 (-36.12 – 213.00)	160.41 (53.43 – 211.73)
Median	0.996	169.21 (-54.69 – 216.22)	166.97 (64.19 – 217.10)
Minimum	0.556	-90.26 (-143.86 – -3.28)	-90.22 (-132.68 – 36.67)
Range	0.030	350.85 (209.54 – 533.25)	381.36 (210.25 – 792.28)
RobustMeanAbsoluteDeviation	0.904	16.96 (8.48 – 49.42)	17.93 (8.16 – 74.10)
RootMeanSquared	0.879	461.72 (268.18 – 515.24)	462.61 (358.71 – 513.24)
Skewness	0.665	-1.78 (-3.37 – 1.80)	-1.54 (-3.54 – -0.15)
Uniformity	0.637	0.05 (0.02 – 0.09)	0.04 (0.01 – 0.09)
Variance	0.706	1773.77 (378.33 – 5850.69)	1651.58 (487.09 – 10482.80)

The data are presented as median values and ranges.
Textural features were calculated using Pyradiomics.
A statistically significant P value is denoted as ** (0.0027 after Bonferroni correction, 0.05 divided by 18, the number of first-order textural features).

Table S3. Univariable Analysis Results for NF-2 Copy Number Loss and S100 Protein Expression

Features	NF-2 Copy Number Loss			S100 Protein Expression		
	**P value**	**OR**	**95 % CI**	**P value**	**OR**	**95 % CI**
10Percentile	0.970	1.00	0.66-1.52	0.351	1.00	0.99-1.01
90Percentile	0.309	0.79	0.51-1.23	0.241	1.01	0.99-1.02
Energy	0.700	0.92	0.61-1.32	0.064	1.00	1.00-1.00
Entropy	0.469	0.85	0.56-1.29	0.620	0.83	0.39-1.74
InterquartileRange	0.181	0.99	0.97-1.00	0.841	1.00	0.98-1.01
Kurtosis	0.635	0.98	0.90-1.07	0.176	0.95	0.88-1.02
Maximum	0.008	0.99	0.98-1.00	0.281	1.00	0.99-1.00
MeanAbsoluteDeviation	0.384	0.99	0.96-1.02	0.644	0.99	0.97-1.02
Mean	0.684	1.00	0.99-1.01	0.323	1.00	0.99-1.02
Median	0.610	0.99	0.98-1.01	0.469	1.00	0.99-1.02
Minimum**	0.539	1.00	0.99-1.02	**0.002**	1,03	1.01-1.04
Range	0.013	0.99	0,99-1.00	0.049	0.99	0.99-1.00
RobustMeanAbsoluteDeviation	0.250	0.98	0.94-1.01	0.823	0.99	0.96-1.03
RootMeanSquared	0.628	0.99	0.98-1.01	0.325	1.00	0.99-1.02
Skewness	0.728	1.09	0.68-1.73	0.166	1.32	0.89-1.96
Uniformity	0.645	215	0.00-1.76 x 10^{12}	0.864	5.77	0.00-3.18 x 10^{9}
Variance	0.295	1.00	1.00-1.00	0.505	1.00	1.00-1.00
Age	0.162	1.02	0.99-1.05	0.659	1.00	0.98-1.03
Growth Pattern	0.303	0.64	0.27-1.49	0.525	1.31	0.57-3.01
Peritumoral Edema	0.160	0.54	0.22-1.27	0.135	0.53	0.23-1.22
Sinus Invasion	0.589	1.30	0.50-3.36	0.738	1.17	0.45-2.99
Hyperostosis	0.662	1.21	0.51-2.89	0.222	0.57	0.23-1.39
Bone Destruction	0.922	0.93	0.21-3.97	0.297	0.42	0.08-2.14
Intratumoral Calcification	0.039	2.56	1.04-6.25	0.328	1.52	0.65-3.54

OR:Odds Ratio
CI: Confidence Interval
“Globose” growth was assigned as 1
“en plaque” growth was assigned as 0
Textural features were calculated using Pyradiomics.
A statistically significant P value is denoted as ** (0.002 after Bonferroni correction, 0.05 divided by 24, the sum of the number of first-order textural features, age and morphological features).

Table S4. Multivariable Analysis Results for NF-2 Copy Number Loss and S100 Protein Expression

Features	NF-2 Copy Number Loss			S100 Protein Expression		
	**P value**	**OR**	**95 % CI**	**P value**	**OR**	**95 % CI**
10Percentile	0.614	1.05	0.87-1.26	0.114	1.14	0.97-1.34
90Percentile	0.209	0.63	0.31-1.29	0.076	1.47	0.96-2.25
Energy	0.187	1.00	1.00-1.00	0.538	1.00	1.00-1.00
Entropy**	0.475	0.03	0.00-34.50 x 10^3	**0.049**	2.16 x 10^{-6}	0.00-0.95
InterquartileRange	0.808	0.94	0.56-1.55	0.167	0.72	0.46-1.14
Kurtosis	0.156	1.42	0.87-2.32	0.133	0.75	0.52-1.08
Maximum**	**0.015**	0.98	0.96-0.99	0.478	1.00	0.99-1.02
MeanAbsoluteDeviation	0.077	3.23	0.88-11.90	0.535	1.32	0.54-3.25
Mean	0.927	1.37	0.00-1160.87	0.929	1.26	0.07-224.61
Median	0.270	0.66	0.32-1.37	0.221	1.30	0.85-1.98
Minimum	0.827	0.99	0.96-1.03	0.960	0.99	0.97-1.03
RobustMeanAbsoluteDeviation	0.747	0.75	0.13-4.17	0.265	2.40	0.51-11.23
RootMeanSquared	0.870	1.67	0.00-822.87	0.699	0.37	0.00-54.97
Skewness	0.102	13.3	0.60-294.99	0.940	1.07	0.16-6.85
Uniformity	0.142	0.00	0.00-2.36 x 10^{23}	0.736	0.00	0.00-4.20 x 10^{70}
Variance	0.882	0.99	0.99-1.01	0.610	0.99	0.99-1.05
Age	0.440	1.01	0.97-1.06	0.783	1.00	0.96-1.04
Growth Pattern**	**0.023**	0.20	0.05-0.79	0.573	1.38	0.44-4.33
Peritumoral Edema	0.334	0.52	0.14-1.93	0.441	0.63	0.20-2.01
Sinus Invasion	0.085	3.99	0.82-19.34	0.524	0.62	0.14-2.66
Hyperostosis	0.697	1.28	0.36-4.62	0.134	0.36	0.09-1.37
Bone Destruction	0.917	0.89	0.11-7.20	0.322	0.31	0.31-3.14
Intratumoral Calcification**	**0.021**	5.39	1.29-22.53	0.252	2.15	0.58-8.03

OR: Odds Ratio
CI: Confidence Interval
“Globose” growth was assigned as 1
“en plaque” growth was assigned as 0
Textural features were calculated using Pyradiomics.
A P value of <=0.05 was considered as statistically significant and is denoted as **.

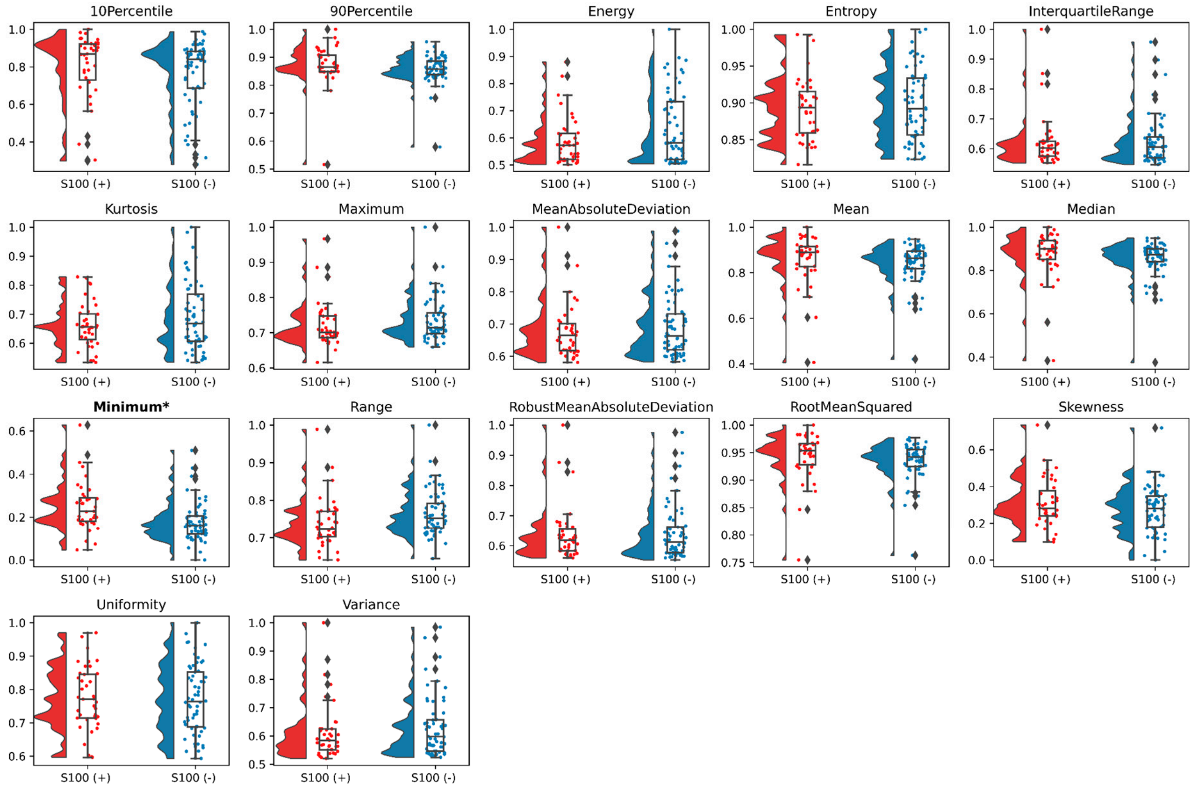

Figure S1. Comparison of textural features for meningiomas with and without S100 protein expression. The data distributions are represented as median and interquartile ranges (IQRs).

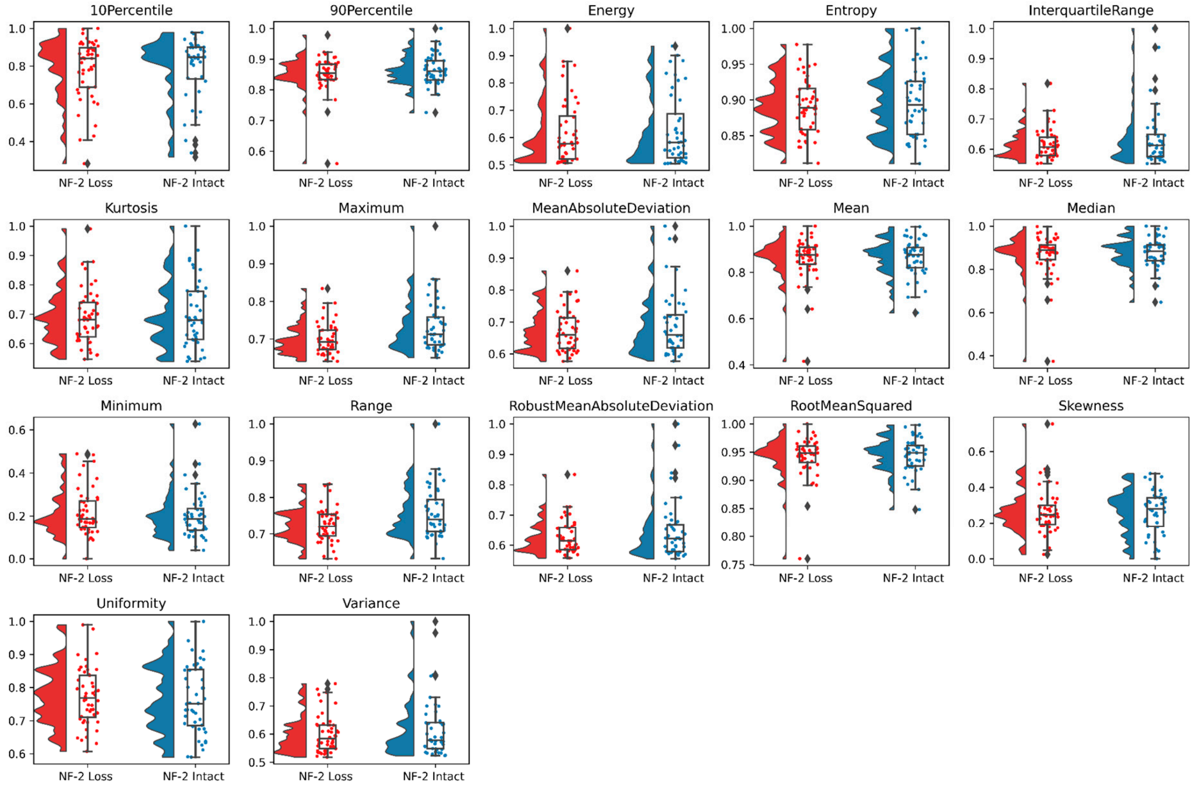

Figure S2. Comparison of textural features for meningiomas with and without NF-2 copy number loss. The data distributions are represented as median and interquartile ranges (IQRs).

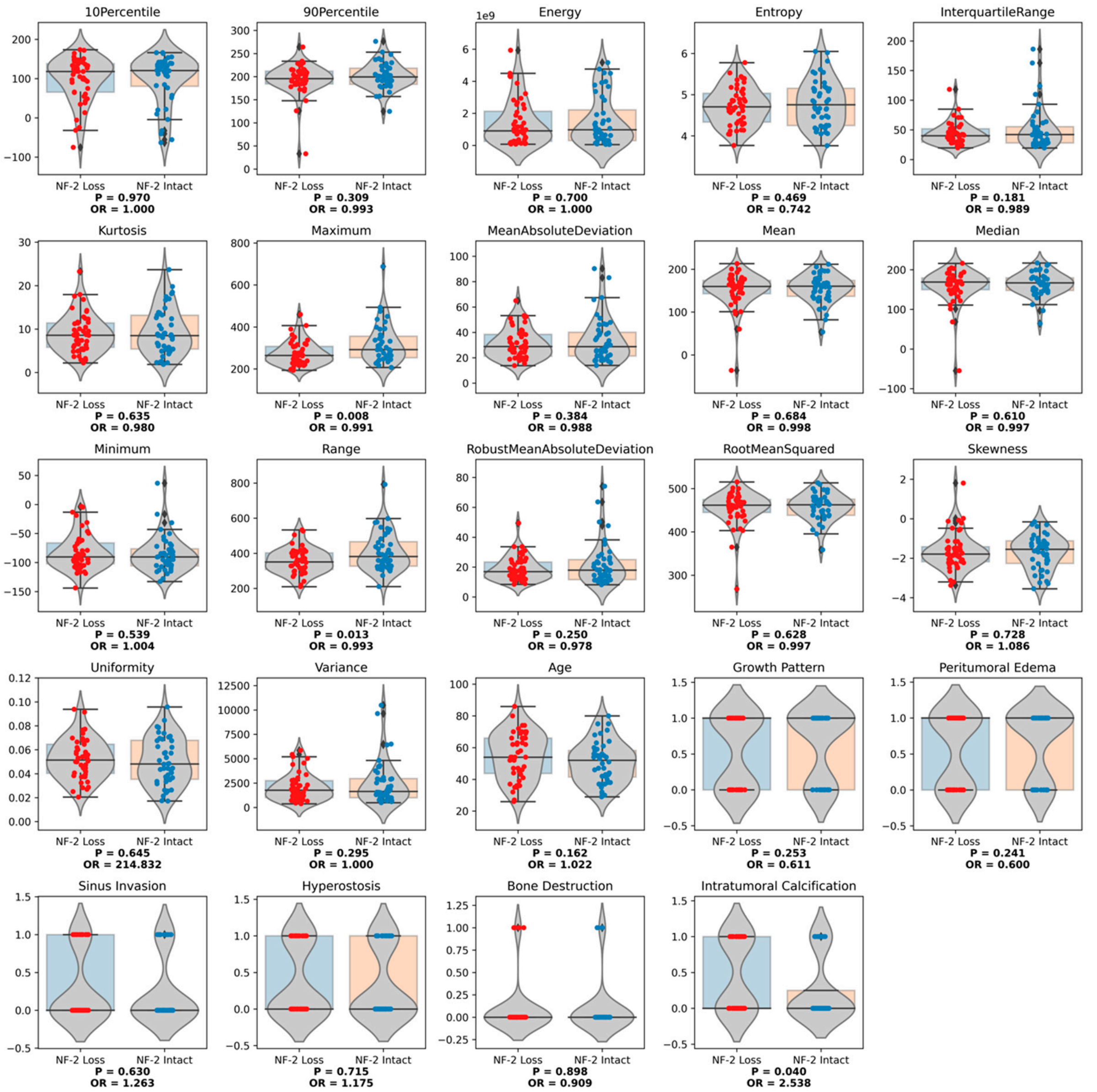

Figure S3. Univariable analysis results for NF-2 copy number loss. The data distributions are represented as median and interquartile ranges (IQRs). "OR" stands for odds ratio and "P" stands for p-value.

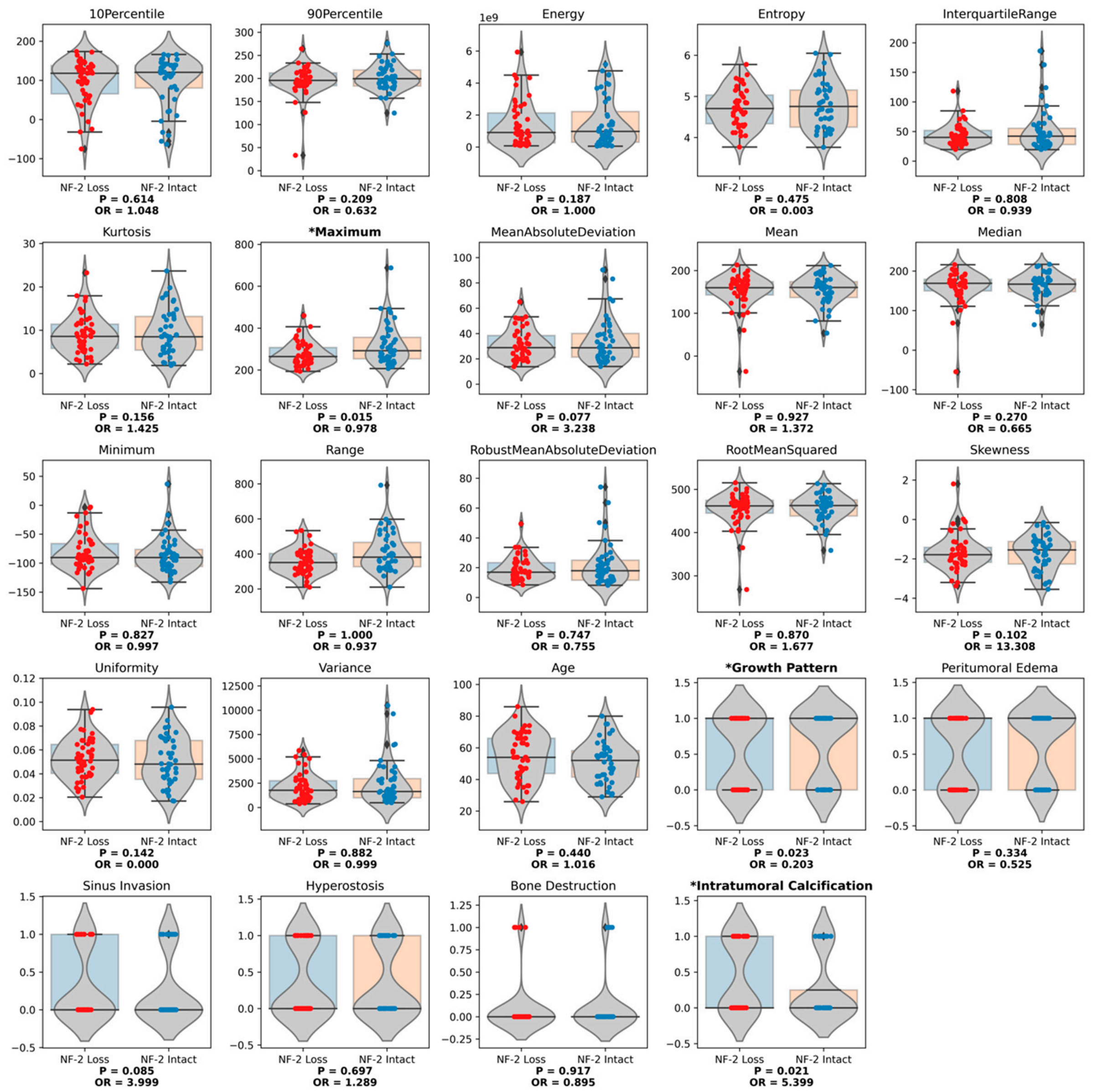

Figure S4. Multivariable analysis results for NF-2 copy number loss. The data distributions are represented as median and interquartile ranges (IQRs). "OR" stands for odds ratio and "P" stands for p-value.

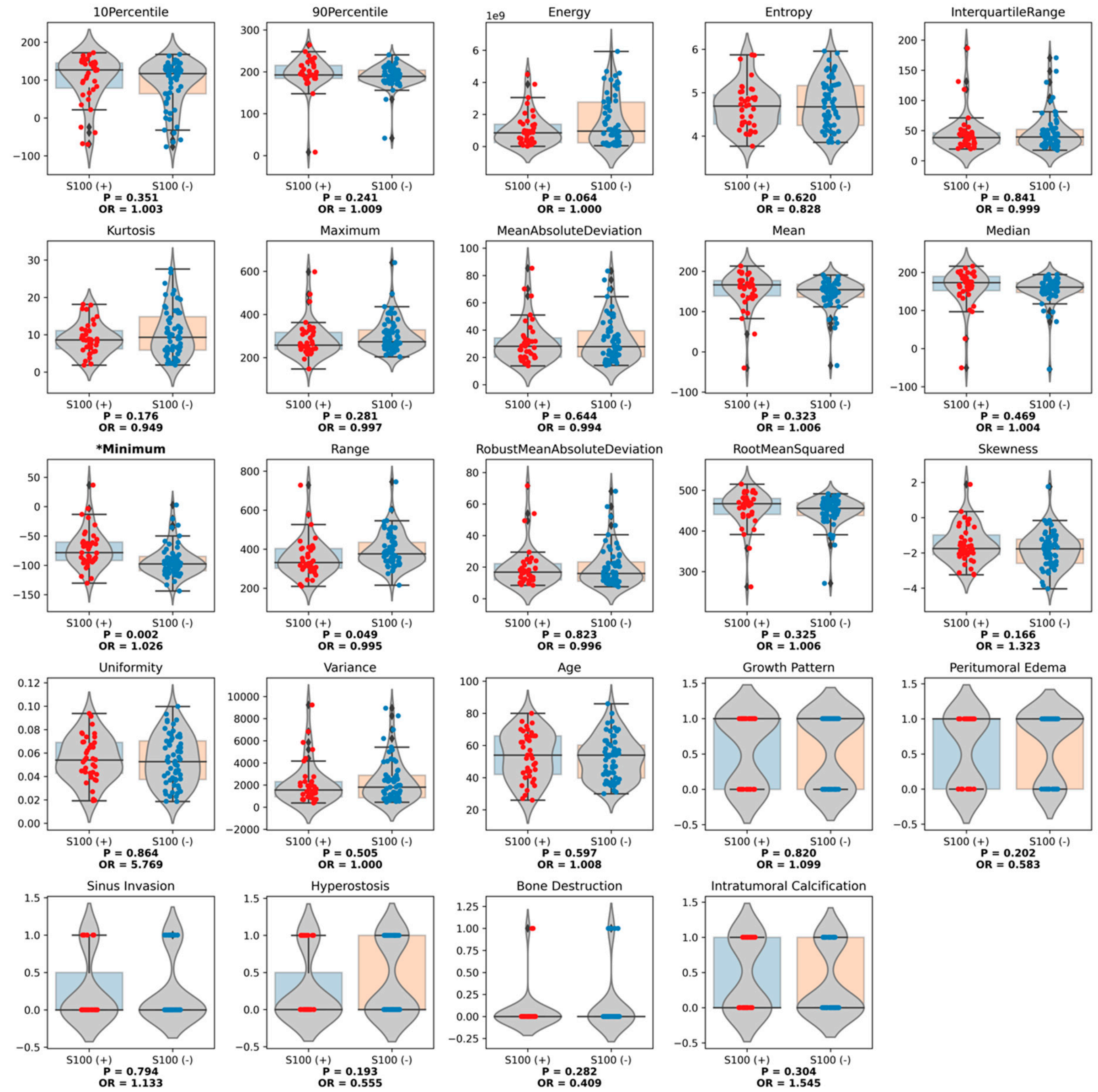

Figure S5. Univariable analysis results for S100 protein expression. The data distributions are represented as median and interquartile ranges (IQRs). "OR" stands for odds ratio and "P" stands for p-value.

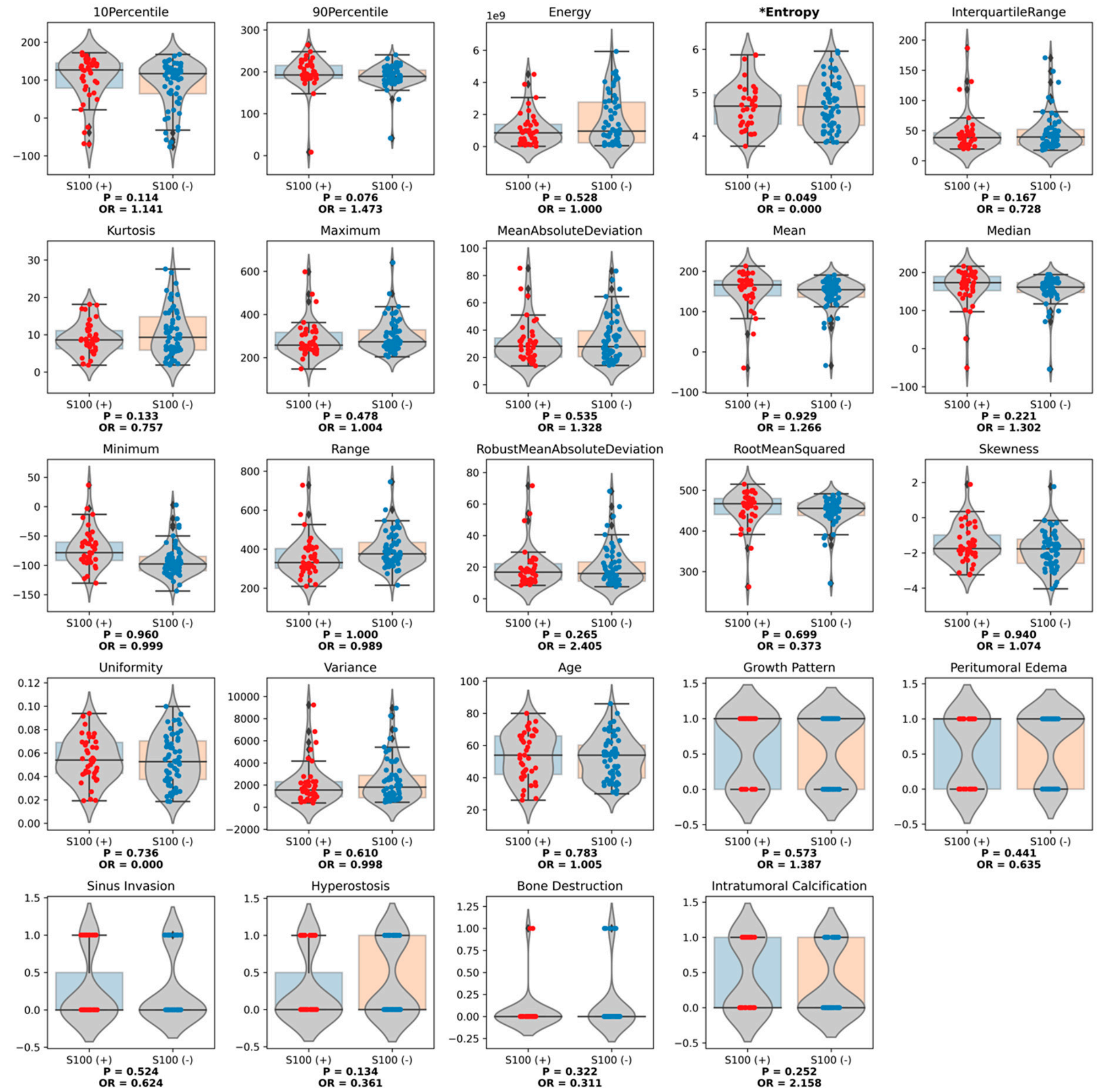

Figure S6. Multivariable analysis results for S100 protein expression. The data distributions are represented as median and interquartile ranges (IQRs). "OR" stands for odds ratio and "P" stands for p-value.

The algorithm parameters applied in conjunction with each multiple classification algorithm employed in this study in section 2.7 are detailed below.

NF-2 Copy Number Loss

"LGBMClassifier(bagging_fraction=0.8, bagging_freq=5, boosting_type='gbdt', class_weight=None, colsample_bytree=1.0, feature_fraction=0.5, importance_type='split', learning_rate=0.3, max_depth=-1,n_min_child_samples=36, min_child_weight=0.001, min_split_gain=0.9, n_estimators=160, n_jobs=-1, num_leaves=60, objective=None, random_state=1574, reg_alpha=0.05, reg_lambda=1e-07, subsample=1.0, subsample_for_bin=200000, subsample_freq=0)"

"AdaBoostClassifier(algorithm='SAMME.R', base_estimator='deprecated', estimator=None, learning_rate=1.0, n_estimators=50, random_state=1574)"

"RandomForestClassifier(bootstrap=False, ccp_alpha=0.0, class_weight='balanced_subsample', criterion='entropy', max_depth=11, max_features=1.0, max_leaf_nodes=None, max_samples=None, min_impurity_decrease=0.002, min_samples_leaf=5, min_samples_split=9, min_weight_fraction_leaf=0.0, n_estimators=80, n_jobs=-1, oob_score=False, random_state=1574, verbose=0, warm_start=False)"

"ExtraTreesClassifier(bootstrap=False, ccp_alpha=0.0, class_weight={}, criterion='entropy', max_depth=1, max_features='sqrt', max_leaf_nodes=None, max_samples=None, min_impurity_decrease=0.001, min_samples_leaf=6, min_samples_split=2, min_weight_fraction_leaf=0.0, n_estimators=20, n_jobs=-1, oob_score=False, random_state=1574, verbose=0, warm_start=False)"

"QuadraticDiscriminantAnalysis(priors=None, reg_param=0.0, store_covariance=False, tol=0.0001)"

"GradientBoostingClassifier(ccp_alpha=0.0, criterion='friedman_mse', init=None, learning_rate=0.3, loss='log_loss', max_depth=7, max_features=1.0, max_leaf_nodes=None, min_impurity_decrease=0.005, min_samples_leaf=4, min_samples_split=7, min_weight_fraction_leaf=0.0, n_estimators=120, n_iter_no_change=None, random_state=1574, subsample=0.45, tol=0.0001, validation_fraction=0.1, verbose=0, warm_start=False)"

"LogisticRegression(C=1.0, class_weight=None, dual=False, fit_intercept=True, intercept_scaling=1, l1_ratio=None, max_iter=1000, multi_class='auto', n_jobs=None, penalty='l2', random_state=1574, solver='lbfgs', tol=0.0001, verbose=0, warm_start=False)"

“GaussianNB(priors=None, var_smoothing=3e-07)”

"DecisionTreeClassifier(ccp_alpha=0.0, class_weight=None, criterion='gini', max_depth=None, max_features=None, max_leaf_nodes=None, min_impurity_decrease=0.0, min_samples_leaf=1, min_samples_split=2, min_weight_fraction_leaf=0.0, random_state=1574, splitter='best')"

"SGDClassifier(alpha=1e-07, average=False, class_weight=None, early_stopping=False, epsilon=0.1, eta0=0.4, fit_intercept=False, l1_ratio=0.9200000001, learning_rate='constant', loss='hinge', max_iter=1000, n_iter_no_change=5, n_jobs=-1, penalty='l1', power_t=0.5, random_state=1574, shuffle=True, tol=0.001, validation_fraction=0.1, verbose=0, warm_start=False)"

"XGBClassifier(base_score=None, booster='gbtree', callbacks=None, colsample_bylevel=None, colsample_bynode=None, colsample_bytree=0.5, device=None, early_stopping_rounds=None, enable_categorical=False, eval_metric=None, feature_types=None, gamma=None, grow_policy=None,

importance_type=None, interaction_constraints=None, learning_rate=0.15, max_bin=None, max_cat_threshold=None, max_cat_to_onehot=None, max_delta_step=None, max_depth=4, max_leaves=None, min_child_weight=3, missing=nan, monotone_constraints=None, multi_strategy=None, n_estimators=20, n_jobs=-1, num_parallel_tree=None, objective='binary:logistic', ...)"
"KNeighborsClassifier(algorithm='auto', leaf_size=30, metric='euclidean', metric_params=None, n_jobs=-1, n_neighbors=35, p=2, weights='uniform')"
"LinearDiscriminantAnalysis(covariance_estimator=None, n_components=None, priors=None, shrinkage=None, solver='svd', store_covariance=False, tol=0.0001)"

S100 Protein Expression

"LGBMClassifier(bagging_fraction=0.8, bagging_freq=5, boosting_type='gbdt', class_weight=None, colsample_bytree=1.0, feature_fraction=0.5, importance_type='split', learning_rate=0.3, max_depth=-1, min_child_samples=36, min_child_weight=0.001, min_split_gain=0.9, n_estimators=160, n_jobs=-1, num_leaves=60, objective=None, random_state=1574, reg_alpha=0.05, reg_lambda=1e-07, subsample=1.0, subsample_for_bin=200000, subsample_freq=0)"
"AdaBoostClassifier(algorithm='SAMME.R', base_estimator='deprecated', estimator=None, learning_rate=1.0, n_estimators=50, random_state=1574)"
"RandomForestClassifier(bootstrap=False, ccp_alpha=0.0, class_weight='balanced_subsample', criterion='entropy', max_depth=11, max_features=1.0, max_leaf_nodes=None, max_samples=None, min_impurity_decrease=0.002, min_samples_leaf=5, min_samples_split=9, min_weight_fraction_leaf=0.0, n_estimators=80, n_jobs=-1, oob_score=False, random_state=1574, verbose=0, warm_start=False)"
"ExtraTreesClassifier(bootstrap=False, ccp_alpha=0.0, class_weight={}, criterion='entropy', max_depth=1, max_features='sqrt', max_leaf_nodes=None, max_samples=None,nmin_impurity_decrease=0.001, min_samples_leaf=6,n, min_samples_split=2, min_weight_fraction_leaf=0.0, n_estimators=20, n_jobs=-1, oob_score=False,nrandom_state=1574, verbose=0, warm_start=False)"
"QuadraticDiscriminantAnalysis(priors=None, reg_param=0.0, store_covariance=False, tol=0.0001)"
"GradientBoostingClassifier(ccp_alpha=0.0, criterion='friedman_mse', init=None, learning_rate=0.3, loss='log_loss', max_depth=7, max_features=1.0, max_leaf_nodes=None, min_impurity_decrease=0.005, min_samples_leaf=4, min_samples_split=7, min_weight_fraction_leaf=0.0, n_estimators=120, n_iter_no_change=None, random_state=1574, subsample=0.45, tol=0.0001, validation_fraction=0.1, verbose=0, warm_start=False)"
"LogisticRegression(C=1.0, class_weight=None, dual=False, fit_intercept=True, intercept_scaling=1, l1_ratio=None, max_iter=1000, multi_class='auto', n_jobs=None, penalty='l2', random_state=1574, solver='lbfgs', tol=0.0001, verbose=0, warm_start=False)"
GaussianNB(priors=None, var_smoothing=3e-07)
"DecisionTreeClassifier(ccp_alpha=0.0, class_weight=None, criterion='gini', max_depth=None, max_features=None, max_leaf_nodes=None, min_impurity_decrease=0.0, min_samples_leaf=1, min_samples_split=2, min_weight_fraction_leaf=0.0, random_state=1574, splitter='best')"

"SGDClassifier(alpha=1e-07, average=False, class_weight=None, early_stopping=False, epsilon=0.1, eta0=0.4, fit_intercept=False, l1_ratio=0.9200000001, learning_rate='constant', loss='hinge', max_iter=1000, n_iter_no_change=5, n_jobs=-1, penalty='l1', power_t=0.5, random_state=1574, shuffle=True, tol=0.001, validation_fraction=0.1, verbose=0, warm_start=False)"
"XGBClassifier(base_score=None, booster='gbtree', callbacks=None, colsample_bylevel=None, colsample_bynode=None, colsample_bytree=0.5, device=None, early_stopping_rounds=None, enable_categorical=False, eval_metric=None, feature_types=None, gamma=None, grow_policy=None, importance_type=None, interaction_constraints=None, learning_rate=0.15, max_bin=None, max_cat_threshold=None, max_cat_to_onehot=None, max_delta_step=None, max_depth=4, max_leaves=None, min_child_weight=3, missing=nan, monotone_constraints=None, multi_strategy=None, n_estimators=20, n_jobs=-1, num_parallel_tree=None, objective='binary:logistic', ...)"
"KNeighborsClassifier(algorithm='auto', leaf_size=30, metric='euclidean', metric_params=None, n_jobs=-1, n_neighbors=35, p=2, weights='uniform')"
"LinearDiscriminantAnalysis(covariance_estimator=None, n_components=None, priors=None, shrinkage=None, solver='svd', store_covariance=False, tol=0.0001)"

Figure S7 and S8 show scree plots for NF-2 copy number loss and S100 protein expression, respectively based on demographic/radiological characteristics and molecular markers using exploratory PCA technique.

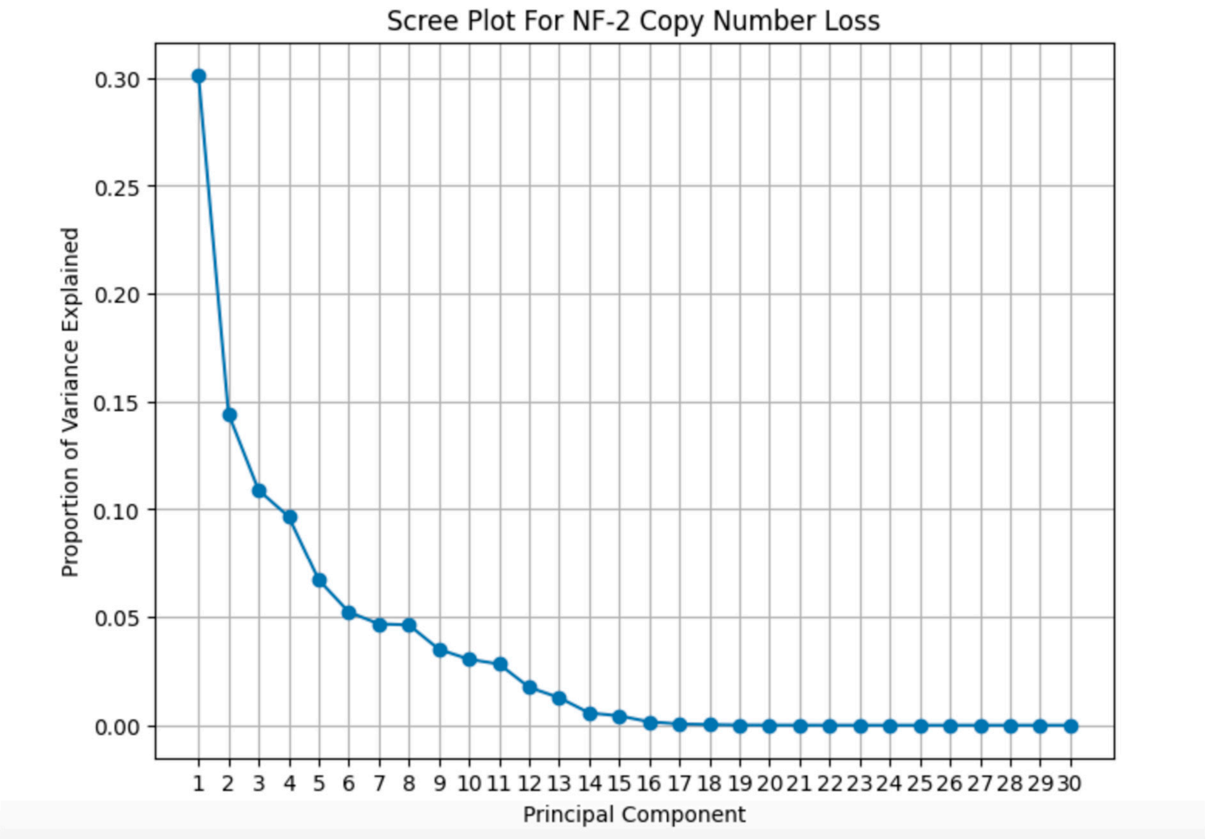

Figure S7. The scree plot for NF-2 copy number loss based on demographic/radiological characteristics and molecular markers using exploratory PCA technique

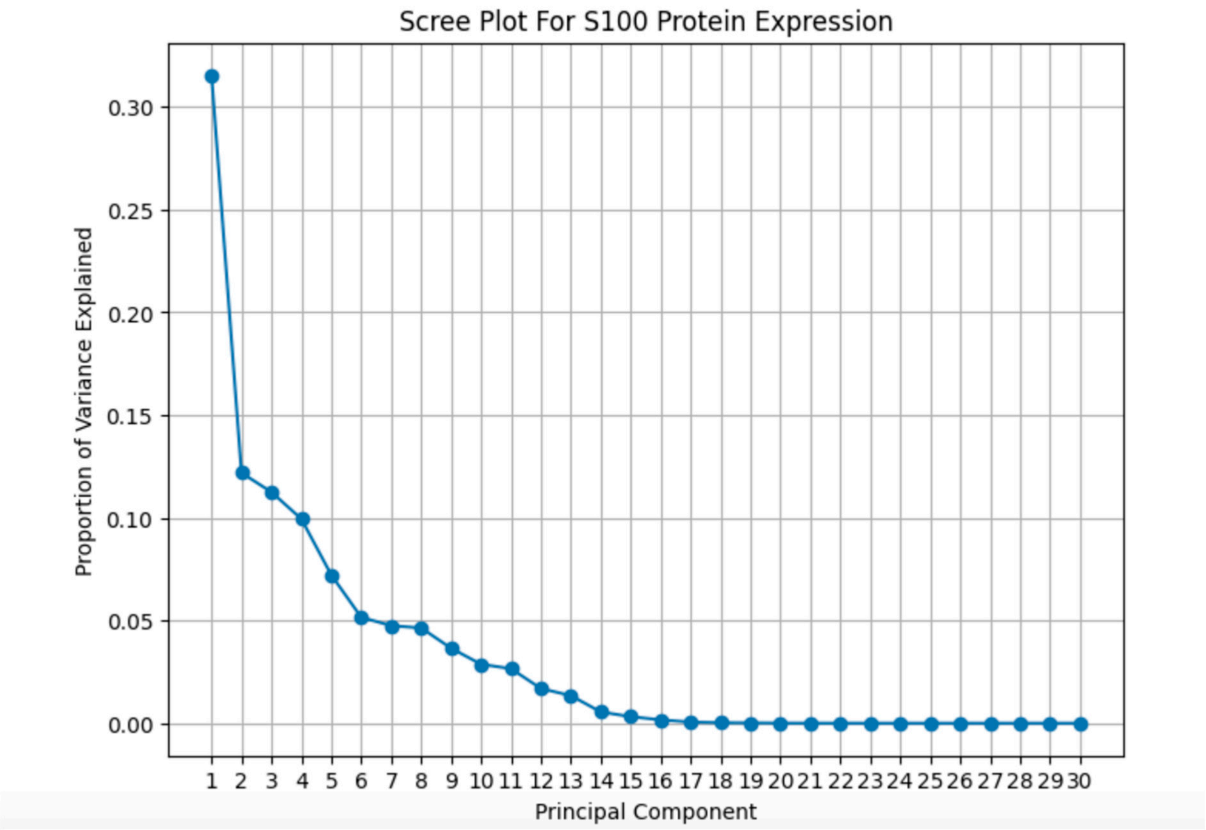

Figure S8. The scree plot for S100 protein expression based on demographic/radiological characteristics and molecular markers using exploratory PCA technique

Figure S9 and S10 illustrate heat maps plotting the association of characteristics with all principal components for the NF-2 copy number loss dataset and the S100 protein expression dataset, respectively. They help identify which characteristics contribute most to each principal component.

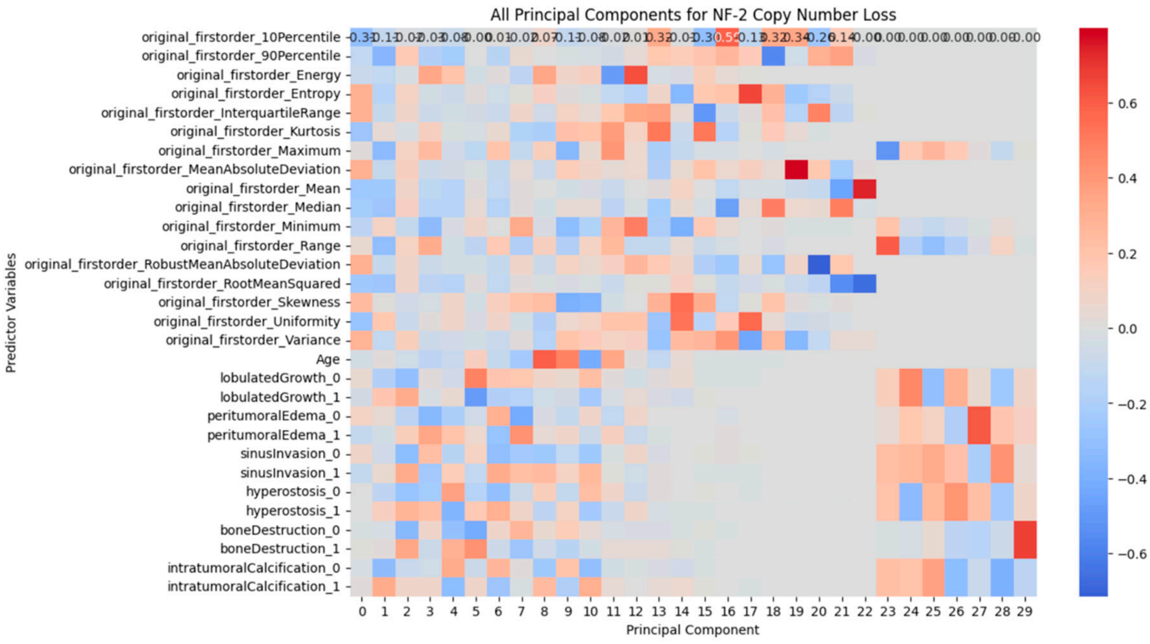

Figure S9. Group associations for all principal components for NF-2 copy number loss

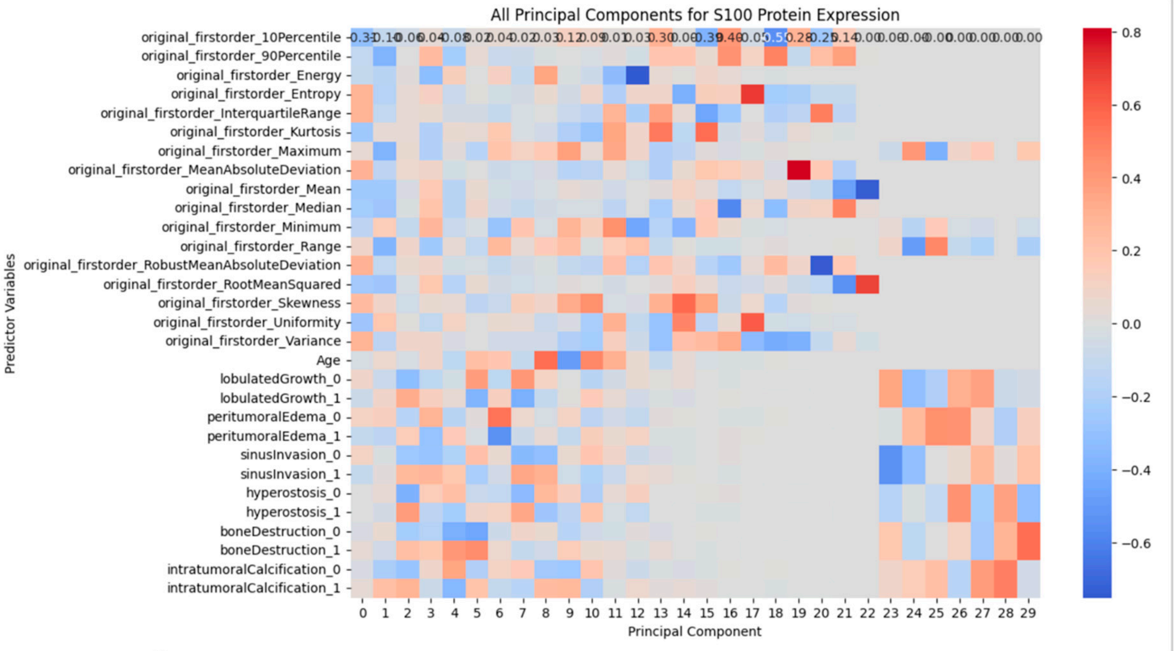

Figure S10. Group associations for all principal components for S100 Protein Expression

For S100 protein expression:

The scree plot revealed that several principal components were strongly associated with demographic and radiological characteristics. Notable features include Root Mean Squared, Peritumoral Edema, Bone Destruction, and Sinus Invasion. Demographic factors like Age were also significant contributors to principal components. Features such as 'Energy' and

'Skewness' emphasized the importance of characteristics in shaping S100 protein expression profiles. Additionally, the 10th Percentile and Entropy of image intensity distribution are significant in understanding S100 protein expression variability.

For NF-2 copy number loss:

Similarly, the scree plot for NF-2 copy number loss analysis reveals principal components strongly associated with demographic and radiological characteristics.
Features like intratumoral calcification, image intensity distribution metrics (e.g., the 90th percentile), and demographic factors such as Age are significant contributors to principal components.
Indicators of tumor characteristics like lobulated growth and peritumoral edema, along with features related to bone destruction and sinus invasion, also contribute significantly to understanding NF-2 copy number loss variability.
Metrics like kurtosis and mean absolute deviation further enhance our understanding of potential molecular markers associated with NF-2 copy number loss.

These findings are derived from group association heatmaps.

S100 Protein Expression Associations:

PC7: peritumoral edema
PC9: Age
PC13, PC14, PC15, PC18, PC19, PC21, PC22, and PC24: These components are associated with various image intensity distribution characteristics, such as energy, kurtosis, skewness, entropy, uniformity, and root mean squared features.
PC30: Bone destruction
PC13: Energy
PC14: Kurtosis
PC15: Skewness
PC18: Entropy and Uniformity
PC19: 10th Percentile Image Intensity

PC21: Interquartile Range and Robust Mean Absolute Deviation
PC22: Root Mean Squared Features
PC24: Sinus Invasion

NF-2 Copy Number Loss Associations:

PC9: Age
PC13, PC14, PC15, PC17, PC18, PC19, PC20, PC21, PC22, PC28, and PC30: These components are associated with various image intensity distribution characteristics and radiological features.
PC13: Energy
PC14: Kurtosis
PC15: Skewness and Uniformity
PC17: 10th Percentile Image Intensity Distributions
PC18: Entropy and Uniformity
PC19: 90th Percentile Image Intensity Distributions
PC20: Mean Absolute Deviation
PC21: Robust Mean Absolute Deviation
PC22: Root Mean Squared of Image Intensity Distributions
PC28: Peritumoral Edema
PC30: Bone Destruction

PCA was also applied to reduce the dimensionality of the deep learning-based features, and scree plots for S100 protein expression and NF-2 copy number loss are provided in Figure S11 and S12, respectively.

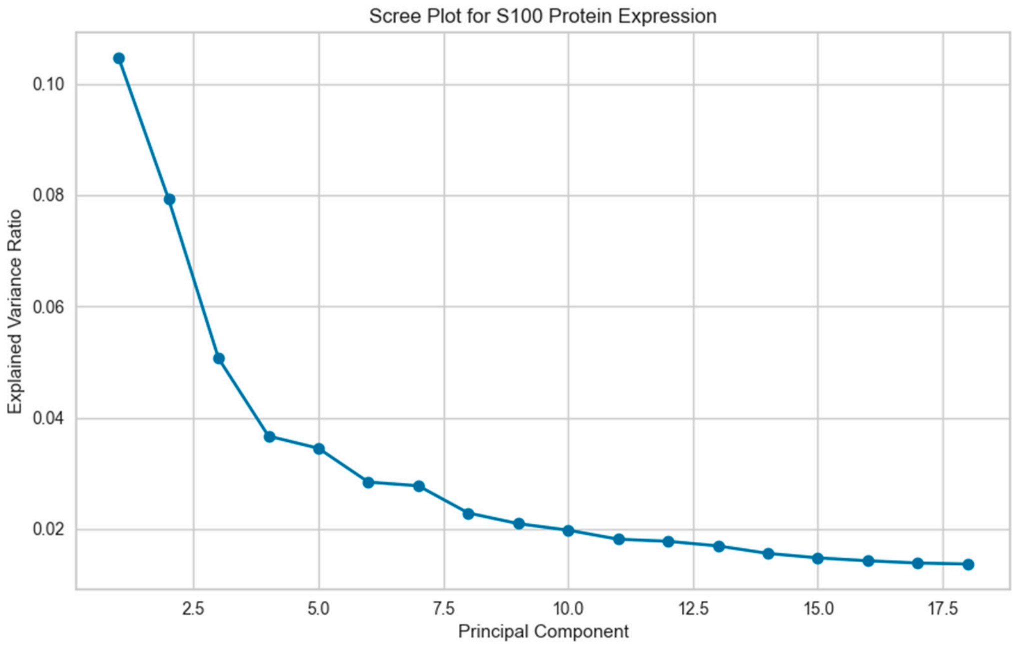

Figure S11. Scree plots for S100 protein expression based on deep learning features

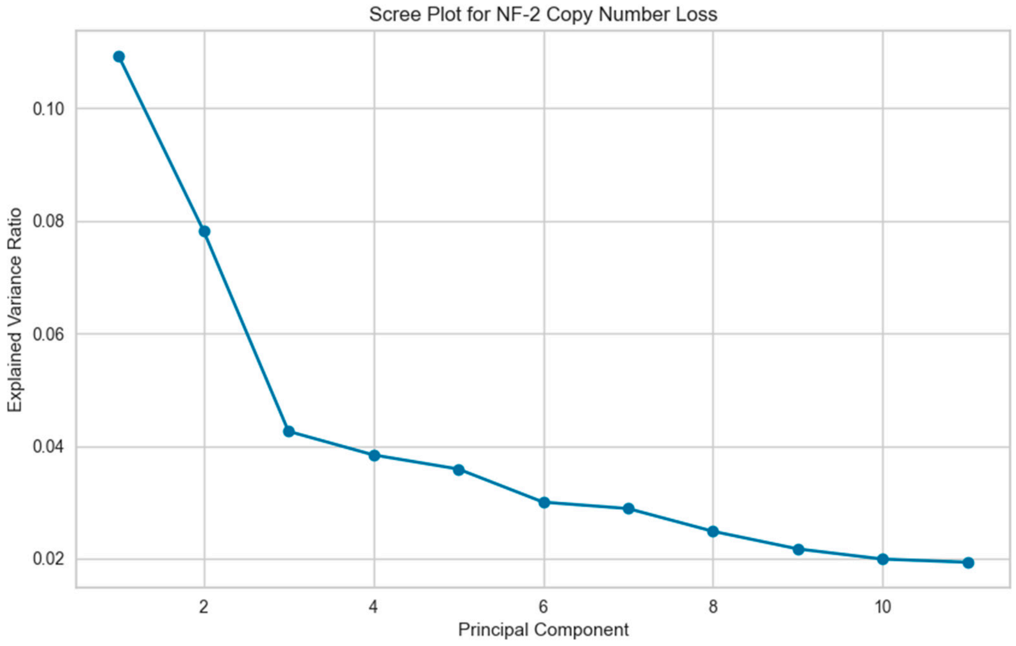

Figure S12. Scree plot for NF-2 copy number loss based on deep learning features

During the feature selection, the feature set was restricted to 18 for the S100 protein expression dataset and 11 for the NF-2 copy number loss dataset, as per the suggestion of exploratory data analysis (EDA). As shown in Figure S11 and S12, 98% variance could be preserved by 10 features in both datasets.

Without using SMOTE in the preprocessing stage, the performance of LGBM decreased. For predicting NF-2 copy number loss, LGBM resulted in an accuracy of 0.63 and an AUC of 0.67 (sensitivity=0.70, specificity=0.55). For predicting S100 protein expression, LGBM resulted in an accuracy of 0.75 and an AUC of 0.76 (sensitivity=0.62, specificity=0.83) .

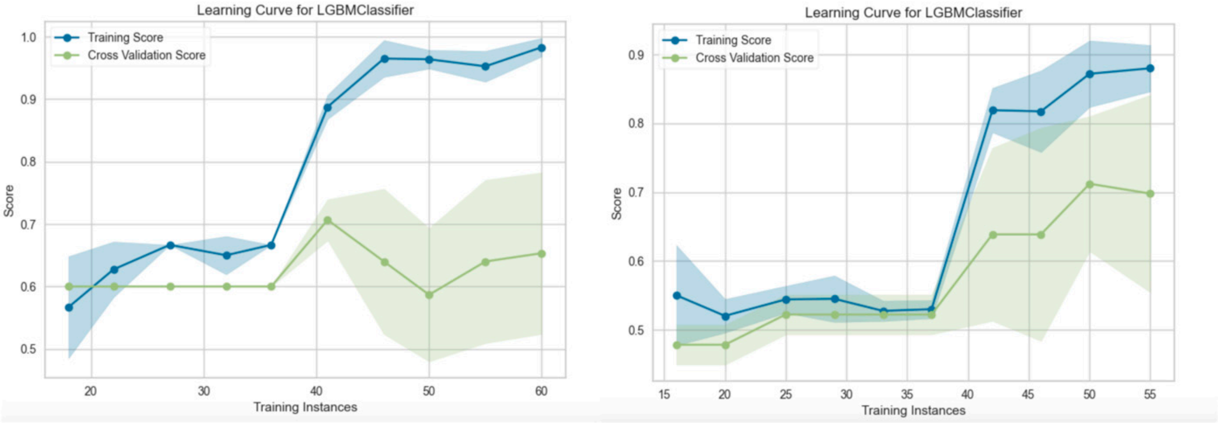

Figure S13. The learning curves and cross-validation scores on the training dataset for the a) S100 protein expression group (number of training instances=75, fold number=5, without SMOTE), and b) NF-2 mutations group (number of training instances=69, fold number=5, without SMOTE). The uncertainty bands in the learning curves depict the standard deviation of the cross-validated scores.

Figure S14 and S15 are feature importance plots. They represent the list of deep learning-based features used to predict NF-2 mutations and S100 protein expression, respectively.

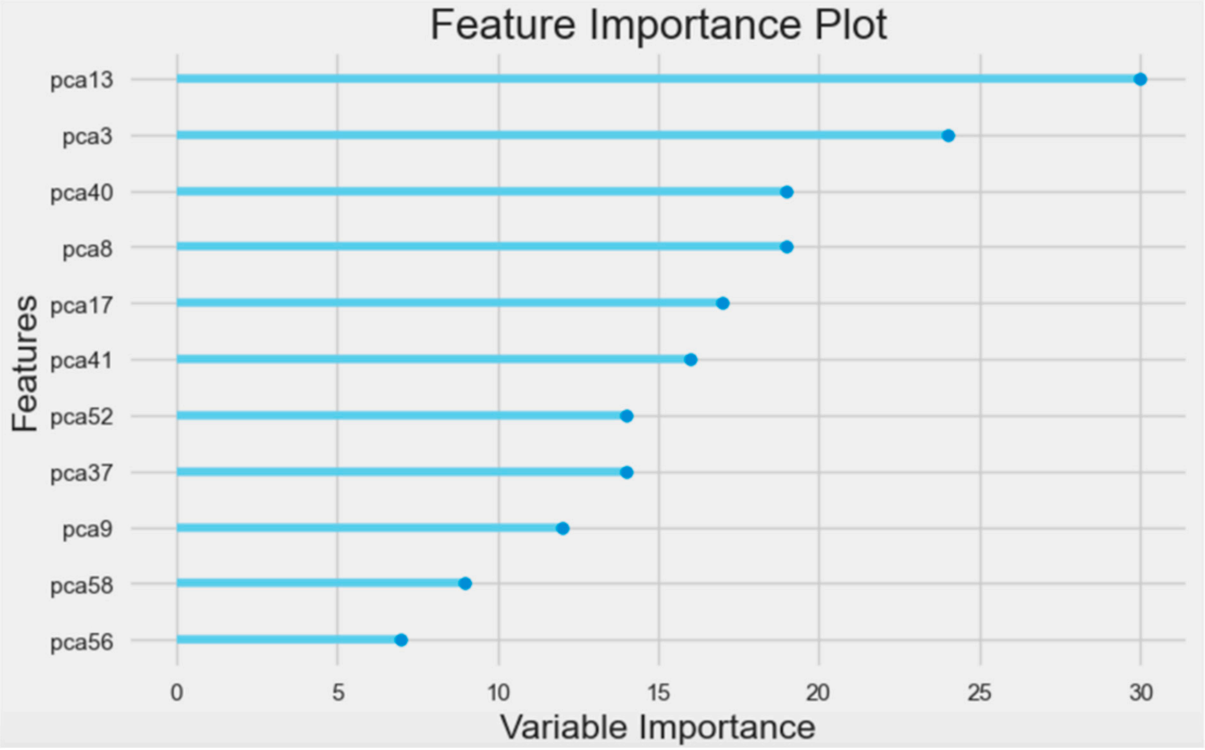

Figure S14. The list of deep learning based features that were used to predict NF-2 mutations

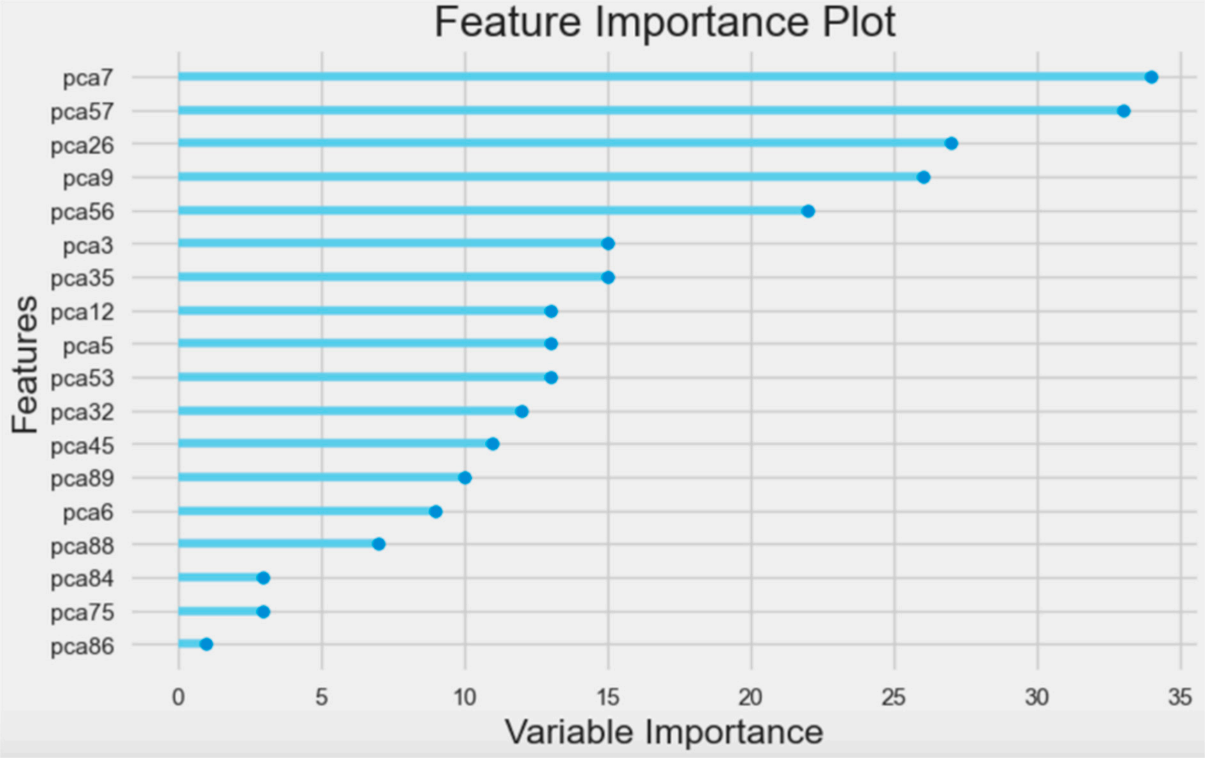

Figure S15. The list of deep learning based features that were used to predict S100 protein expression
